# Morphology and phylogeny reveal two new species and a new geographical record of *Agaricus* sect. *Rubricosi* (Agaricales) from West Bengal, India

**DOI:** 10.1038/s41598-025-08102-2

**Published:** 2025-07-12

**Authors:** Entaj Tarafder, Samantha C. Karunarathna, Arun Kumar Dutta, Abdallah M. Elgorban, Feng-Hua Tian, Krishnendu Acharya

**Affiliations:** 1 Department of Plant Pathology, Agriculture College, Guizhou University, Guiyang, 550025 Guizhou People’s Republic of China; 2 Molecular and Applied Mycology and Plant Pathology Laboratory, Department of Botany, University of Calcutta, 35, Ballygunge Circular Road, Kolkata, West Bengal 700019 India; 3 Center for Yunnan Plateau Biological Resources Protection and Utilization, College of Biology and Food Engineering, Qujing Normal University, Qujing, 655011 People’s Republic of China; 4 Department of Botany, Gauhati University, Gopinath Bordoloi Nagar, Jalukbari, Guwahati, Assam 781014 India; 5 Department of Botany and Microbiology, College of Science, King Saud University, 11451 Riyadh, Saudi Arabia

**Keywords:** *Agaricus* subgenus *Pseudochitinia*, *Agaricaceae*, Basidiomycota, Phylogeny, Taxonomy, Molecular biology, Plant sciences

## Abstract

Two new species (*Agaricus tumlingensis*, and *A*. *philippei*) and one new record (*A*. *kunmingensis*) belong to the sect. *Rubricosi* of the subg. *Pseudochitonia*, are described from India based on morphological and molecular sequence (nrITS and LSU rDNA) data. *Agaricus tumlingensis* is distinguished by its medium to large-sized basidiomata, brown to dark brown squamulose pileus, ellipsoid to elongate basidiospores with a range value of 5.5–7.5 × 3.5–4.0 μm, and cheilocystidia measuring 15–34 × 9–17 μm. *Agaricus philippei* has a dark brown appressed squamulose pileus, ellipsoid basidiospores with a range value of 4.5–5.1 × 2.5–3.0 μm, and cheilocystidia measuring 11–21 × 7–14 μm. Detailed morphological descriptions, illustrations, field photographs of the collected basidiomata, comparisons with morphologically similar species, and phylogenetic analysis results based on the nrITS and nrLSU sequence data are provided.

## Introduction

*Agaricus* L. is a large genus with high species diversity, comprising nearly 600 species described across the globe^1,2^. The species of *Agaricus* are saprotrophic and can be found in a variety of tropical and temperate climates around the world^3–7^. The genus *Agaricus* is characterized by small to large, fleshy fruiting bodies with white to variously colored pileus, free lamellae that are white or pink when young, become brown to dark brown at maturity, presence of an annulus on the stipe, pileipellis a cutis of cylindrical hyphae^4,5,8–12^. The genus has long been a topic of interest to researchers worldwide due to its taxonomic diversity and frequent discoveries of new species. However, the genus is easily recognized in the field based on morphological characteristics, but distinguishing between species can be challenging due to intraspecific variability^4,13,14^.

Currently, six subgenera of the genus *Agaricus* are widely accepted; these are *Agaricus* subg. *Agaricus* L., *A*. subg. *Flavoagaricus* Wasser, *A*. subg. *Minoriopsis* Linda J. Chen, L.A. Parra, Callac, Angelini & Raspé, *A*. subg. *Minores* (Fr.) R.L. Zhao & Moncalvo, *A*. subg. *Pseudochitonia* Konrad & Maubl., and *A*. subg. *Spissicaules* (Heinem.) R.L. Zhao & Moncalvo and 27 Sects.^4,6,12,15–17^. *Agaricus* subg. *Conioagaricus*^8^ has an epithelium pileipellis, which is completely different from that of species in the other subgenera; thus, *A*. subg. *Conioagaricus* was suggested to be relocated to other genera^12,18^. Unfortunately, in the absence of available molecular data, *A*. subg. *Conioagaricus* has not been studied molecularly within the new taxonomic system proposed by Zhao et al.^12^. *Agaricus* subg. *Lanagaricus*^8^ has been proven a heterotypic synonym of *A*. subg. *Pseudochitonia*^12^.

Specifically, the species of *A*. subg. *Pseudochitonia* consists of 14 sections, *A*. sect. *Bivelares* (Kauffman) L.A. Parra, *A*. sect. *Bohusia* (L.A. Parra) L.A. Parra & R.L. Zhao, *A*. sect. *Brunneopicti* Heinem., *A*. sect. *Chitonioides* Romagn., *A*. sect. *Crassispori* R.L. Zhao, *A*. sect. *Cymbiformes* M.Q. He & R.L. Zhao, *A*. sect. *Flocculenti* J. Chen, K.D. Hyde & R.L. Zhao, *A*. sect. *Hondenses* R.L. Zhao & L.A. Parra, *A*. sect. *Nigrobrunnescentes* K.P. Peterson, Desjardin & Hemmes, *A*. sect. *Rubricosi* R.L. Zhao, *A*. sect. *Sanguinolenti* Jul. Schäff & F.H. Møller ex L. A. Parra, *A*. sect. *Trisulphurati* Heinem., *A*. sect. *Xanthodermatei* Singer and *A*. sect. *Catenulati* S. Hussain & Al-Sadi. Zhao et al.^12^ demonstrated that *Agaricus* sect. *Rubricosi* is a monophyletic group, as determined by molecular phylogenetic analyses of Asian taxa. Currently, this section comprises four known species viz. *Agaricus magnivelaris* Pegler, described from tropical America (Pegler, 1983); *A*. *variabilocolor* R.L. Zhao, described from Thailand; *A*. *kunmingensis* R.L. Zhao, designated as the type species, and *A*. *dolichopus* R.L. Zhao, both described from China^12^. Until now, no additional species have been formally described within this section. Members of sect. *Rubricosi* are characterized by a pileus and stipe surface that may or may not discolor reddish-brown upon bruising, a negative Schäffer’s reaction (aniline × nitric acid), an indistinct or slightly reddish-brown reaction with KOH, an indistinct, pleasant, or phenol-like odor, a double annulus that is superior, membranous, and often floccose on the lower side, and pyriform cheilocystidia^12^.

To date, a total of 104 species of *Agaricus* have been reported from India^19–22^. However, most of these species were identified solely based on morphology, following descriptions from European or North American literature^23^which necessitates a revision under modern taxonomic concepts. Notably, no species from the sect. *Rubricosi* have been documented in India, whereas two species within the subg. *Pseudochitonia* have been recognized based on modern taxonomic approaches^20,22^. Addressing this gap, the present study provides a comprehensive taxonomic evaluation of species belonging to *Agaricus* sect. *Rubricosi* and subg. *Pseudochitonia*, integrating both morphological and molecular data.

Within the framework of the authors’ ongoing research on *Agaricus* in West Bengal, Eastern India^1,20,21^ this study reports two new taxa in *A*. sect. *Rubricosi* and one new geographical record based on morphological and molecular phylogenetic analyses.

## Materials and methods

### Sample collection and morphological characterization

Fresh basidiomata were collected from several grasslands and forested areas in West Bengal, eastern India, during the monsoon months of June–September (2018–2021). The Canon EOS 1200D (Canon, India) digital camera was used to take photos of fresh basidiomata in their natural habitat. The fresh basidiomata were subjected to chemical reactions as described by Chen et al.^35^. The specimens were systematically described in terms of their morphology following Largent et al.^24^. For the notation of colors, we referred to the Methuen Handbook of color^25^. The basidiomata were then dried in a field dryer at 40–45°C^26^.

For microscopic characterization, thin free hand sections from multiple basidiomata were taken and mounted in 5% KOH and stained with 1% Congo red. The notation [30, 1, 1] indicates that the measurements were made on 30 basidiospores in one sample from one collection, and the abbreviations X_m_, the arithmetic mean of the spore length by the spore width (± standard deviation), Q for the quotient of length and width, and Q_m_ for the mean of Q values (± standard deviation). Dried voucher specimens were deposited in the Calcutta University Herbarium (CUH) for future reference.

### DNA extraction, polymerase chain reaction (PCR) amplification, and sequencing

**Fig. 1:**
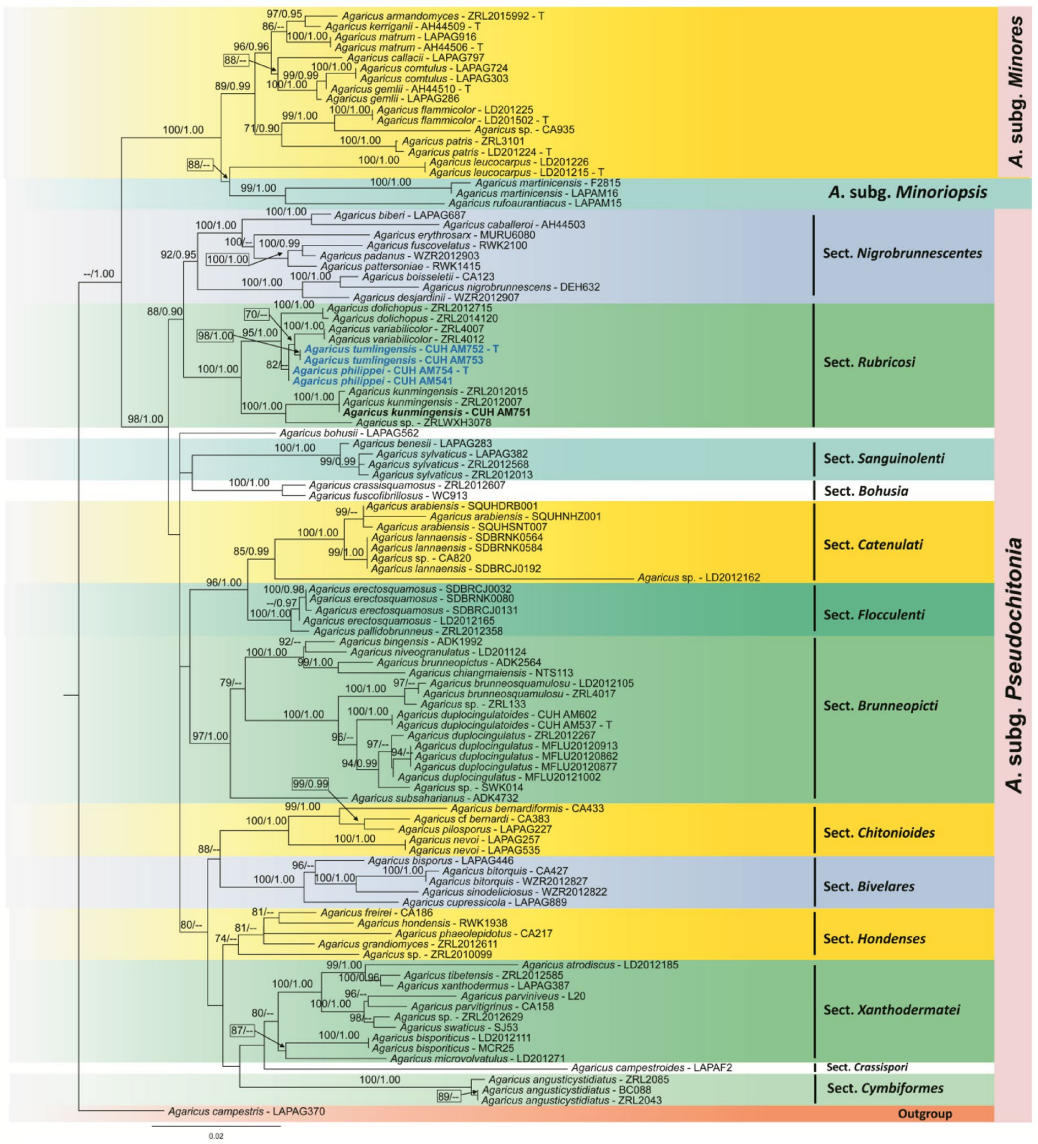
Phylogram generated from maximum likelihood (ML) analysis based on the dataset of nrITS and LSU rDNA sequences. One taxon, *A*. *campestris* (LAPAG370) of *Agaricus* subg. *Agaricus* is used as the outgroup taxa following Zhao et al.^12^ and Tarafder et al.^1^. The best-scoring RAxML tree with a final likelihood value of -*InL* = 9242.755121 is presented. Bootstrap (BS) support values received from ML and Bayesian posterior probabilities (BPP) analyses values equal to or greater than 70% and 0.95 are given above the nodes (ML values on the left side of ‘/’ in regular font and PP values on the right side of ‘/’ in italics). Type specimen sequences of the newly generated species are represented in bold blue. Voucher specimen numbers for the sequences are indicated in the tree after the taxon name.

Genomic DNA was extracted from dried specimens using E.Z.N.A.^®^ Fungal DNA Mini Kit (Omega Bio-Tek, Inc., Norcross, USA) following the manufacturer’s protocol with minor modifications for fungal material and also followed other *Agaricus* related studies^1,27,28^. The PCR amplification of the nrITS and LSU rDNA regions was done following Tarafder et al.^21^ using primer pairs ITS1 and ITS4^29^ and LR0R and LR3^30^, respectively. The amplified PCR products were visualized via UV light through 1% Agarose gel electrophoresis, stained with ethidium bromide, and sequencing of the amplified PCR product was performed by AgriGenome Labs Pvt. Ltd., Kerala, India, using the same primer pairs described above. The newly generated sequences were checked with BioEdit v. 7.2.5^31^ and deposited in the NCBI GenBank (https://www.ncbi.nlm.nih.gov/genbank; Table 1; Fig. 1) nucleotide database for future reference.

**Table 1:** Names, voucher numbers, geographic origin, and corresponding GenBank accession numbers for the NrDNA ITS sequences of the taxa used in the phylogenetic analyses.

Sl. No.	Name of the species	Voucher number	GenBank accession numbers		Geographical origin	References
			nrITS	LSU rDNA		
1	*A. angusticystidiatus*	BC088	MG888054	–	Thailand	^7^
2	*A. angusticystidiatus*	ZRL2085	KT951434	MG835413	Thailand	^7^
3	*A. angusticystidiatus* T	ZRL2043	JF691553	MG835412	Thailand	^7^
4	*A. arabiensis*	SQUH–DRB001	OM971854	–	Oman	^32^
5	*A. arabiensis*	SQUH-SNT007	OM971855	OM971859	Oman	^32^
6	*A. arabiensis*	SQUH-NHZ001	OM971852	OM971853	Oman	^32^
7	*A. armandomyces* T	ZRL2015992	KX684860	KX684882	China	^33^
8	*A. atrodiscus*	LD2012185	KT284912	KT951473	Thailand	^34^
9	*A. benesii*	LAPAG283	JF797179	–	Spain	^4^
10	*A. bernardiformis*	CA433	KT951321	KT951467	Spain	^12^
11	*A. biberi*	LAPAG687	KM657919	KR006614	Hungary	^12^
12	*A. bingensis*	ADK1992	KJ540954	–	Benin	^35^
13	*A. bisporiticus*	LD2012111	KJ575611	KT951507	Thailand	^11^
14	*A. bisporiticus* T	MCR25	KJ575608	–	Pakistan	^11^
15	*A. bisporus*	LAPAG446	KM657920	KR006611	Spain	^36^
16	*A. bitorquis*	CA427	KT951320	KT951491	China	^12^
17	*A. bitorquis*	WZR2012827	KM657916	–	China	^36^
18	*A. bohusii*	LAPAG562	KM657928	KR006613	Spain	^37^
19	*A. boisseletii*	CA123	DQ182531	–	France	^3^
20	*A. brunneopictus*	ADK2564	JF514518	**–**	Benin	^4^
21	*A. brunneosquamulosu*	LD2012105	KJ540968	**–**	Thailand	^35^
22	*A. brunneosquamulosu*	ZRL4017	JF691549	–	Thailand	^4^
23	*A. caballeroi*	AH-44,503	KJ575605	–	Spain	^38^
24	*A. callacii*	LAPAG797	KF447899	KX083984	Spain	^39^
25	*A. campestris*	LAPAG370	KM657927	KP739803	Spain	^37^
26	*A. campestroides*	LAPAF2	JF727842	–	Togo	^4^
27	*A. bernardi*	CA383	KT951319	KT951469	France	^12^
28	*A. chiangmaiensis*	NTS113	JF514531	**–**	Thailand	^4^
29	*A. comtulus*	LAPAG724	KT951332	KT951448	Spain	^6^
30	*A. comtulus*	LAPAG303	KU975078	KX083986	Spain	^6^
31	*A. crassisquamosus*	ZRL2012607	KT951376	KT951510	China	^12^
32	*A. cupressicola*	LAPAG889	KT951334	KT951465	Italy	^12^
33	*A. desjardinii*	WZR2012907	KM657901	KT951474	China	^36^
34	*A. dolichopus*	ZRL2012715	KT951382	KT951502	China	^12^
35	*A. dolichopus*	ZRL2014120	KT951433	–	China	^12^
36	*A. duplocingulatoides*	CUH AM602	MH511804	–	India	^20^
37	*A. duplocingulatoides*	CUH AM537	MH511677	–	India	^20^
38	*A. duplocingulatus*	ZRL2012267	KT951368	KT951504	Thailand	^12^
39	*A. duplocingulatus*	MFLU:2012_0913	KJ540963	–	Thailand	^35^
40	*A. duplocingulatus*	MFLU:2012_0862	KJ540967	–	Thailand	^35^
41	*A. duplocingulatus*	MFLU:2012_0877	KJ540965	–	Thailand	^35^
42	*A. duplocingulatus*	MFLU:2012_1002	KJ540961	–	Thailand	^35^
43	*A. erectosquamosus*	SDBR-CJ0032	MW255804	MW255803	Thailand	^40^
44	*A. erectosquamosus*	SDBR-NK0080	MW255805	–	Thailand	^40^
45	*A. erectosquamosus*	SDBR-CJ0131	MW255807	–	Thailand	^40^
46	*A. erectosquamosus*	LD2012165	KT951338	KT951509	Thailand	^12^
47	*A. erythrosarx*	MURU6080	JF495068	–	Australia	^41^
48	*A. flammicolor*	LD201225	KU975115	KX084010	Thailand	^6^
49	*A. flammicolor* T	LD201502	KU975114	KX084009	Thailand	^6^
50	*A. freirei*	CA186	DQ185553	–	France	^3^
51	*A. fuscofibrillosus*	WC913	AY484684	–	USA	^42^
52	*A. fuscovelatus*	RWK2100	KJ577973	–	USA	^14^
53	*A. gemlii*	LAPAG286	KU975079	KX083988	Spain	^6^
54	*A. gemlii* T	AH44510	KF447891	KX083989	Spain	^6^
55	*A. grandiomyces*	ZRL2012611	KM657879	KR006624	China	^37^
56	*A. hondensis*	RWK1938	DQ182513	–	California	^3^
57	*A. kerriganii* T	AH44509	KF447893	KX083999	Spain	^39^
58	*A. kunmingensis*	ZRL2012015	KT951361	KT951506	China	^12^
59	*A. kunmingensis* T	ZRL2012007	KT951427	–	China	^12^
60	***A. kunmingensis***	**CUH AM751**	**OM279500**	**PV341363**	**India**	**This study**
61	*A. lannaensis*	SDBR-NK0564	MW255657	MW255674	Thailand	^40^
62	*A. lannaensis*	SDBR-NK0584	MW255738	MW262926	Thailand	^40^
63	*A. lannaensis*	SDBR-CJ0192	MW255680	–	Thailand	^40^
64	*A. leucocarpus*	LD201226	KU975102	KX083982	Thailand	^6^
65	*A. leucocarpus* T	LD201215	KU975101	KX083981	Thailand	^6^
66	*A. martinicensis*	F2815	JF727855	KX084032	Martinique (France)	^4^
67	*A. martinicensis*	LAPAM16	KX671699	KX671709	Dominican Republic	^6^
68	*A. matrum*	LAPAG916	KU975080	KX083990	Spain	^6^
69	*A. matrum* T	AH44506	KF447896	KX083991	Spain	^6^
70	*A. microvolvatulus*	LD201271	KJ575614	KT951508	Thailand	^11^
71	*A. nevoi*	LAPAG257	KM657922	KR006606	Spain	^36^
72	*A. nevoi*	LAPAG535	KT951330	–	Spain	^12^
73	*A. nigrobrunnescens*	DEH632	JX308267	–	Hawaii, USA	^43^
74	*A. niveogranulatus*	LD201124	KJ540959	–	Thailand	^35^
75	*A. padanus*	WZR2012903	KM657903	KR006616	China	^12^
76	*A. pallidobrunneus*	ZRL2012358	KT951370	KT951471	China	^12^
77	*A. parviniveus*	L20	MK101027	–	Pakistan	^16^
**78**	***A. philippei***	**CUH AM754**	**OM654926**	**PV455289**	**India**	**This study**
**79**	***A. philippei***	**CUH AM541**	**PQ396192**	–	**India**	**This study**
80	*A. parvitigrinus*	CA158	AY899267	–	France	^44^
81	*A. patris*	ZRL3101	JF691544	KX084013	Thailand	^4^
82	*A. patris* T	LD201224	KU975118	KX084012	Thailand	^6^
83	*A. pattersoniae*	RWK1415	AY943974	–	USA	^3^
84	*A. phaeolepidotus*	CA217	DQ185552	–	France	^3^
85	*A. pilosporus*	LAPAG227	KT951425	–	Spain	^12^
86	*A. rufoaurantiacus*	LAPAM15	KT951313	KX671708	Dominican Republic	^12^
87	*A. sinodeliciosus*	WZR2012822	KM657907	KT951518	China	^36^
88	*A. sp.*	ZRL2012629	KM657890	KR006627	China	^37^
89	*A. lannaensis*	CA820	JF727861		Thailand	^4^
90	*A. malakandesis*	LD2012162	KT951337	KT951493	Thailand	^12^
91	*A. sp.*	ZRL2010099	KT951349	KT951479	China	^12^
92	*A. sp.*	SWK014	KT951342	KT951482	Malaysia	^12^
93	*A. sp.*	ZRL 133	KT951344	KT951505	Thailand	^12^
94	*A. sp.*	ZRLWXH3078	KT951389	KT951464	China	^12^
95	*A. sp.*	CA935	KU975085	KX084036	Thailand	^6^
96	*A. subsaharianus*	ADK4732	JF440300	**–**	Burkina Faso	^45^
97	*A. swaticus*	SJ53	KY741894	–	Pakistan	^16^
98	*A. sylvaticus*	LAPAG382	KM657929	KR006608	Spain	^37^
99	*A. sylvaticus*	ZRL2012013	KT951360	KT951500	China	^12^
100	*A. sylvaticus*	ZRL2012568	KT951371	–	China	^12^
101	*A. tibetensis*	ZRL2012585	KM657895	KR006633	China	^37^
**102**	***A. tumlingensis***	**CUH AM752**	**OM654924**	**PV341365**	**India**	**This study**
**103**	***A. tumlingensis***	**CUH AM753**	**OM654925**	**PV341366**	**India**	**This study**
104	*A. variabilicolor*	ZRL4007	KT951439	–	Thailand	^12^
105	*A. variabilicolor*	ZRL4012	KT951440	–	Thailand	^12^
106	*A. xanthodermus*	LAPAG387	KM657923	KR006609	Spain	^37^

Details of newly generated sequences in the present work are provided in bold. ‘–’ means no relevant genetic information.

### Sequence acquisition and dataset preparation

Newly amplified sequences were used for BLASTn searches in the NCBI GenBank nucleotide database. Based on the BLASTn results, closely related sequences of the taxa with zero E-values were selected for constructing the dataset. Additionally, the sequences used in earlier studies on *Agaricus*^12,16^ were also downloaded from the database for preparing the final dataset (GenBank numbers shown in Table 1; Fig. 1).

### Sequence alignment and phylogenetic analyses

The sequences of nrITS were aligned with MAFFT v.7.427^46^ online tool https://www.ebi.ac.uk/Tools/msa/mafft/, and the alignments were improved manually wherever necessary using MEGA v.7.0^47^. Finally, the dataset comprised 106 nrITS sequences, distributed over 86 ingroup taxa belonging to all 14 available sections of the *Agaricus* subg. *Pseudochitonia* and 21 taxa belonging to three closely related subgenera (*A*. subg. *Agaricus*, *A*. subg. *Minoriopsis* & *A*. subg. *Minores*), including one outgroup taxon of *A*. subg. *Agaricus* viz. *A*. *campestris* (LAPAG370) following Zhao et al.^12^ and Tarafder et al.^1^.

A model test was performed with the concatenated dataset to choose the best-fit model. For this purpose, jModeltest 2.1.10 v20160303^48^ was used at the CIPRES web portal^49^. The lowest BIC score of 14912.371 indicated that GTR + I + G was the best-fit model for the given dataset in maximum likelihood (ML) analysis.

ML bootstrapping (MLBS) analyses were performed using RAxML-HPC2 v.8.2.1250, with the specified model parameters obtained from jModelTest2 on the CIPRES web portal. Bootstrap statistics were calculated from 1000 bootstrapping replicates.

Bayesian analyses (BA) were done with MrBayes v.3.2.2^50^ under a GTR + I + G model. Markov chains were run for 1.0 × 10^6^ generations, saving a tree at every 100th generation and setting all remaining parameters to their default values. The initial 25% of trees recovered were excluded as burn-in, and the remaining trees were then used to estimate the posterior probability (PP) values of the group.

## Results

### Phylogenetic analyses

The phylogenetic analyses were conducted using the combined datasets of nrITS and LSU rDNA sequences (Fig. 1). All 106 sequences were aligned, and both ends were trimmed to create a dataset of 647 nucleotides that included 234 parsimony-informative characters, 370 constants (proportion = 0.7789988), and 43 parsimony-uninformative characters. Bayesian analyses (BA) reached a standard deviation of split frequencies of 0.0030 after 1,000,000 generations, and the credible sets contained 12,001 trees, excluding the initial 3,000 trees as the burn-in. The phylogenetic tree obtained from ML and MrBayes analyses showed an almost identical topology. Therefore, the tree obtained from the best ML analysis (*InL* = -9242.755121) is displayed in the present manuscript (Fig. 1), and support values were recovered from ML (MLBS ≥ 70%), and BA (PP ≥ 0.95) are reported.

MLBS and PP values support many of the terminal nodes but fail to recover deeper nodes with high statistical support. Members of *A*. subg. *Pseudochitonia* appear in different clades within the phylogenetic tree (Fig. 1). The 86 ingroup *Agaricus* sequences are distributed across 13 available sections of the *Agaricus* subgenus. *Pseudochitonia* cluster together and this cluster was strongly supported by both ML and Bayesian analyses (98% MLBS and 1.00 PP). The newly generated sequences of the Indian specimens *A. tumlingensis*, and *A. philippei* fall into a clade together with the other well-representative members of *A*. sect. *Rubricosi*, confirming their systematic position within that section.

Within sect. *Rubricosi*, two newly generated nrITS and LSU rDNA sequences of *Agaricus tumlingensis* (CUH AM752) are sister to *Agaricus variabilicolor* (ZRL4007), but they differ in having moderately supported ML statistical values (70% MLBS) and unsupported Bayesian statistical values. The other newly described taxon, *Agaricus philippei* (CUH AM754), sister to *Agaricus dolichopus* (ZRL2014120), was previously deposited as a sequence from Thailand and determined as a type with moderate statistical support values. However, the Indian collection of *Agaricus kunmingensis* (CUH AM751) clusters together with the two Chinese collections along with the type species (ZRL2012007) with full support values (MLBS 100%, 1.00 PP), indicating that the Indian specimens are conspecific with the Chinese collections.

A comparison made from the alignment of the entire nrDNA ITS sequences of the four samples of the two new species, *Agaricus tumlingensis* sp. nov., and *Agaricus philippei* sp. nov., differs at 13 positions from the four well-represented samples of *Agaricus* sect. *Rubricosi* available in the GenBank nucleotide database (Table 2).

**Table 2:** Comparison of the entire NrDNA ITS region (647 nucleotides) between the newly amplified four sequences of *Agaricus tumlingensis* sp. Nov., *A*. *philippei* sp. Nov. (in bold font), and one new record (*A*. *kunmingensis*) with sequences of closely related taxa of *Agaricus* subg. *Pseudochitonia* sect. *Rubricosi*.

Name of the taxa	Nucleotide differences in the ITS 1 + 2 alignment (647 nts)												
	6	23	47	103	106	108	225	303	498	508	543	592	647
*A. kunmingensis* (CUH AM751)	C	C	T	T	A	T	C	A	C	G	G	G	C
*A. kunmingensis* (ZRL2012007)	C	C	T	T	A	A	C	A	C	G	G	A	C
***A. tumlingensis*** **(CUH AM752)**	**T**	**T**	**T**	**C**	**A**	**T**	**C**	**C**	**T**	**G**	**A**	**G**	**G**
***A. tumlingensis*** **(CUH AM753)**	**T**	**T**	**T**	**C**	**A**	**T**	**C**	**C**	**T**	**G**	**A**	**G**	**G**
***A***. ***philippei*** **(CUH AM754)**	**C**	**C**	**C**	**C**	**G**	**T**	**T**	**T**	**T**	**G**	**A**	**G**	**A**
***A***. ***philippei*** **(CUH AM541)**	**C**	**C**	**C**	**C**	**G**	**T**	**T**	**T**	**T**	**G**	**A**	**G**	**A**
*A. dolichopus* (ZRL2014120)	C	T	C	T	A	A	C	A	G	A	G	G	C
*A. variabilicolor* (ZRL4007)	T	T	C	T	G	A	T	C	T	G	A	A	C
*A.* sp. (ZRLWXH3078)	C	T	T	T	G	T	T	A	C	G	G	G	T

### Taxonomy

*Agaricus kunmingensis* R.L. Zhao Fungal Diversity: 78: 275 (2016) Figs. 2A–B and 3.

*Morphological characteristics* Basidiomata small to medium sized, agaricoid. Pileus 40–60 mm diam., convex when young, becoming plano-convex with a flat umbo at the center; surface dry, covered by squamules, dense surrounding the disc, triangular with recurved tips, brown (7F4) near the umbo, brown (7E5) to reddish-orange (10D4) towards margin; margin straight and sometimes appendiculate. Context up to 5 mm broad, at first white, then quickly turning slightly rubescent at the centre on exposure, finally light brown (7D7) after a few minutes. Lamellae 3–5 mm broad, free, crowded with three series of lamellulae, brown to dark brown (7F7), unchanging on bruising; edge concolorous. Stipe 75–80 × 3–5 mm, equal, hollow, surface smooth or slightly fibrillose, white (1A1) when young, turn light brown (6D5-6) on bruising. Annulus ca. 5 mm in diam., membranous, entire or torned, pendant, white (1A1), upper surface smooth and lower surface floccose with brown (7E6-8) patches at the margin. Odour indistinct.

*Macrochemical reactions* KOH reaction positive, color turns brown (7E6) on the pileus surface; Schäffer’s reaction on the pileus surface negative.

*Microscopical characteristics* Basidiospores [60/2/1] (5.0–)5.2–6.2(–7.0) × (2.5–)3.0–3.2(–3.5) µm, [Xm = 5.7 ± 0.8 × 3.1 ± 0.3 μm, Q = 1.4–1.8, Qm = 1.6 ± 0.1], ellipsoid to elongate, brown with KOH, smooth, thick-walled, without germ pore. Basidia 14–17 × 5–7.5 μm, clavate, hyaline, smooth, tetrasterigmate, sterigma 2.5–3 μm long. Basidioles 10–16 × 5–7 μm, clavate, hyaline, smooth. Cheilocystidia 12–27 × 9–15 μm, abundant, pyriform, broadly clavate, smooth, hyaline. Pleurocystidia absent. Pileipellis a cutis composed of hyphae 3.5–13 μm broad, slightly constricted at the septa, contain light brown pigments. Stipitipellis hyphae 4–7 μm broad, hyaline, thin-walled, smooth. Annulus composed of hyphae 4–9 μm broad, hyaline, branched, cylindrical.

*Habit*, *habitat*, *and distribution*: solitary to gregarious, scattered, on humus soil among mixed forests of *Alnus* and *Millettia*. Distributed in the subtropical region of India and the tropical region of Southwest China^12^.

*Specimen examined*: INDIA: West Bengal, Kalimpong District, Lingsey Village, 27°10’12.1"N, 88°40’13.26"E, alt. 1105.0 m asl., 28 April 2018, E. Tarafder & J. Tamang, Entaj-10/2018, CUH AM751. GenBank: CUH AM751 - ITS = OM279500; LSU = PV341363.

*Notes*: *Agaricus kunmingensis* was originally described based on a collection made in the Yunnan Province of China^12^. The Indian collections exhibit almost identical morphological features to the type description, except for a larger pileus size (40–60 mm vs. 20–35 mm) and longer basidiospores (5.0–7.0 μm, Q mean 1.6 μm vs. 4.1–5.3 μm, Q mean 1.5). Based on molecular phylogenetic analyses, the present collection showed 99% sequence identity with *A*. *kunmingensis* (ZRL2012015) from China^12^confirming its geographic similarity to *A*. *kunmingensis*.

**Fig. 2:**
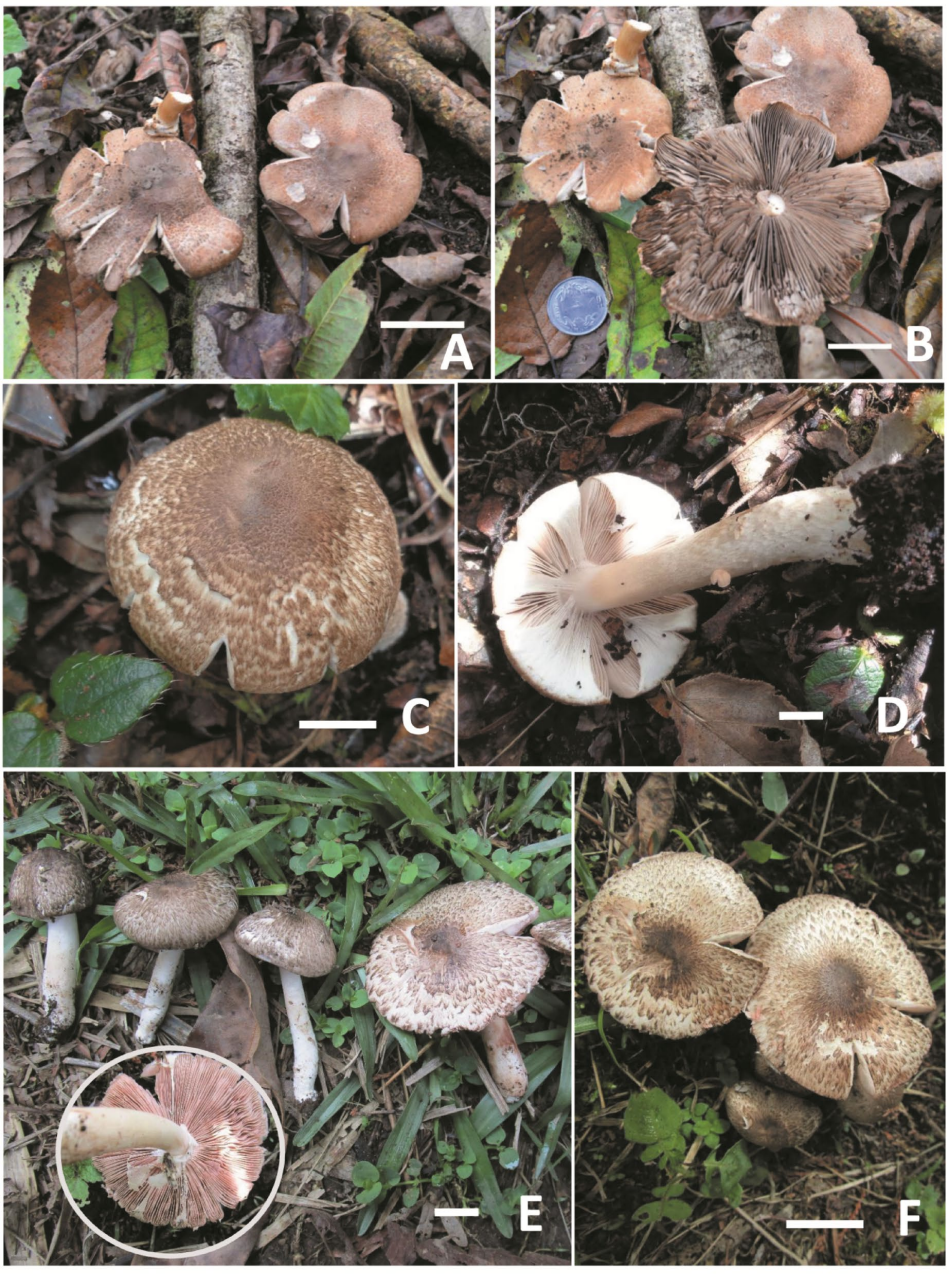
*Agaricus* sect. *Rubricosi.* Field photographs of the basidiomata of (**A**–**B**) *Agaricus kunmingensis*. (**C**–**D**) *Agaricus tumlingensis*. (**E**–**F**) *Agaricus philippei*. Scale bars: (**A**) = 20 mm, (**B**) = 10 mm, (**C**–**E**) = 20 mm, (**F**) = 10 mm. Photographed by: E. Tarafder.

**Fig. 3:**
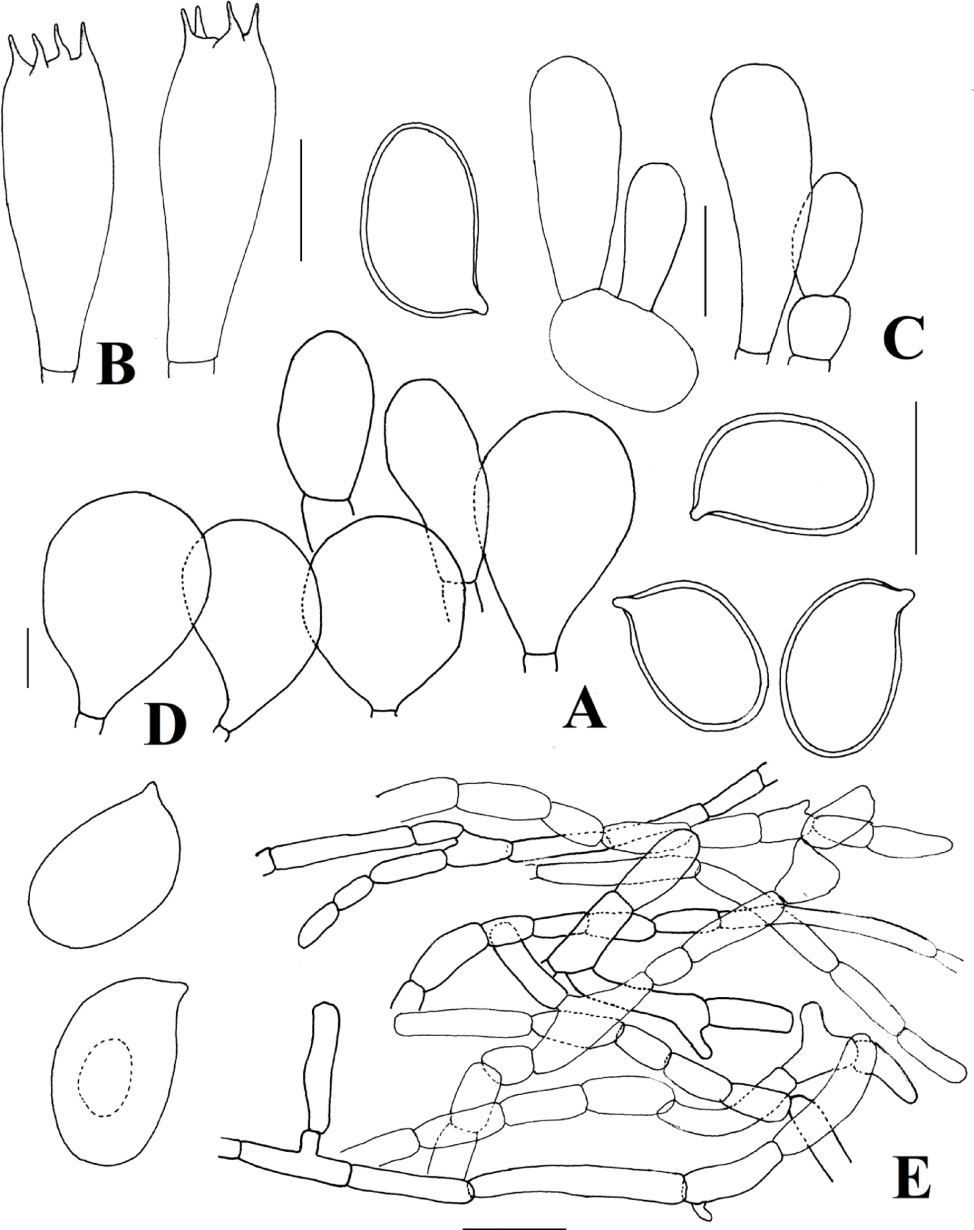
*Agaricus kunmingensis*. (**A**) Basidiospores (**B**) basidia (**C**) basidioles (**D**) cheilocystidia (**E**) pileipellis hyphae. Scale bars: (**A**–**D**) = 5 μm, (**E**) = 10 μm. Drawings by: E. Tarafder.

There are no significant morphological and molecular differences observed in the Indian collection compared to the type specimen from China, confirming our collection as *A*. *kunmingensis*. This is the first report of its occurrence outside the type locality (Figs. 2A-B and 3).

**Fig. 4:**
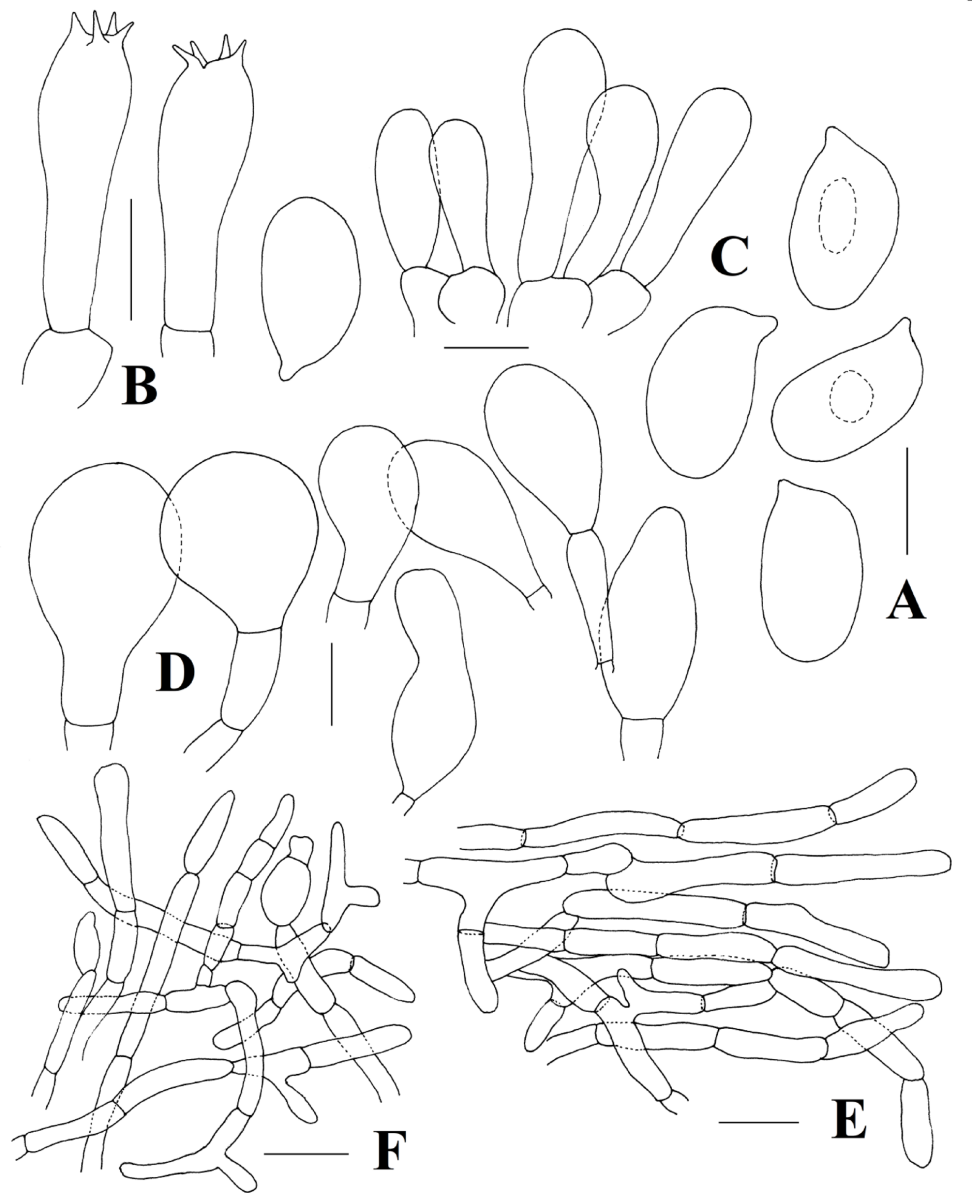
*Agaricus tumlingensis*. (**A**) Basidiospores (**B**) basidia (**C**) basidioles (**D**) cheilocystidia (**E**) pileipellis hyphae (**F**) annulus hyphae. Scale bars: (**A**) = 5 μm, (**B**) = 10 μm, (**C**)  = 5 μm, (**D**) = 10 μm, (**E**–**F**) = 20 μm. Drawings by: E. Tarafder.

*Agaricus tumlingensis* E. Tarafder & K. Acharya, *sp. nov*. Figures 2C–D and 4.

Index Fungorum IF902821.

*Typification*: INDIA: West Bengal, Darjeeling District, Tumling Village, 27°1′53.61′′ N, 88°4′6.54′′ E, alt. 2918.0 m asl., 5 June 2017, E. Tarafder, Entaj-004 (holotype CUH AM752). GenBank: CUH AM752 - ITS = OM654924; LSU = PV341365.

*Diagnosis* Differs from *Agaricus variabilicolor* by the presence of brown to dark brown fibrillose squamules on the pileus surface, longer basidiospores 5.5–7.5 × 3.5–4.0 μm and Q = 1.7 on average, and larger cheilocystidia (15–34 × 9–17 μm) and distinctive nrITS and LSU rDNA sequences and position in the phylogram.

*Etymology* The specific epithet “*tumlingensis*” (Lat.) refers to the Tumling Village, a subtropical region in highland eastern India, where the holotype was collected.

*Morphological characteristics* Basidiomata medium sized. Pileus 33–86 mm diam., convex to broadly convex with a small umbo, surface fibrillose to squamules, fibrils/squamules dense at the disc, spreading either throughout the entire cap or sometimes slightly less dense from the middle towards margin, grayish-brown in center (6-7F3), elsewhere brown (7E5) on a white background, margin entire, often rimose. Context 2–4 mm thick at the centre, gradually thinner towards margin, white Lamellae 3–5 mm broad, free, pinkish white (11A2) when young, greyish brown (5E1) on maturity, concolorous, moderately crowded with 1–2 series of lamellulae. Stipe 54–102 × 5–7 mm, gradually broader towards base (14–17 mm), central, cylindrical, white (1A1), slightly brownish (6E3) when extreme old, turning pastel red (7A4) to greyish red (7B4) on bruising, base covered by dense squamules, gradually lesser towards apex when young, basal squamules white, elsewhere light brownish on a white background, squamules disappear upon maturity and the entire surface remains white, context narrowly hollow to stuffed. Annulus superous, simple, thin, fragile, attached at the pileus margin, sometimes leaving broken traces on the stipe, white (1A1) to cream and evanescent. Odour indistinct.

*Macrochemical reactions* KOH positive, color turns slightly reddish brown on the pileus and stipe surface. Schäffer’s reaction was negative on the pileus surface.

*Microscopical characteristics* Basidiospores [60/2/1] (4.7–)5.5–7.5(–8.5) × (2.5–)3.5–4.0(–5.0) µm, [Xm = 6.5 ± 1.2 × 3.7 ± 0.6 μm, Q = 1.5–1.9, Qm = 1.7 ± 0.1], ellipsoid to elongate, light brown to brown, smooth, thick-walled, Basidia 18–25 × 6–9 μm, clavate to cylindrical, hyaline, smooth, tetra-sterigmatic, sterigmata 0.5–1 μm long. Basidioles 12–15 × 5–6 μm, clavate, hyaline, thin-walled, smooth. Cheilocystidia 15–34 × 9–17 μm, pyriform, narrowly utriform, broadly clavate, smooth, hyaline. Pleurocystidia absent. Pileipellis a cutis composed of hyphae 6–9.5 μm broad, slightly constricted at the septa, contain light brown pigments. Stipitipellis hyphae 5–6 μm broad, hyaline, thin-walled, smooth. Annulus composed of hyphae 6.5–10 μm broad, hyaline, branched, cylindrical, slightly bulbous apex, 6–9 μm wide.

*Habit*, *habitat*, *and distribution*: solitary, scattered, on humus-mixed soil among mixed vegetation. Distributed in a subtropical region of India.

*Additional specimen examined*: INDIA: West Bengal, Darjeeling District, Tumling Village, 27°1′53.58′′ N, 88°4′8.32′′E, alt. 2917.0 m asl., 12 July 2018, *E. Tarafder*, Entaj-126, CUH AM753. GenBank: CUH AM753 - ITS = OM654925, LSU = PV341366.

*Notes*: *Agaricus tumlingensis* is characterized by brown-colored appressed fibrils; lamellae crowded; stipe base turns pastel red to greyish red on bruising and covered by dense white squamules; a fragile, superous annulus; ellipsoid to elongate basidiospores (4.7–8.5 × 2.5–5.0 μm, Q mean of 1.7); and presence of pyriform to narrowly utriform or broadly clavate cheilocystidia (15–34 × 9–17 μm, Figs. 2C-D); and phylogenetic analyses based on nrITS and LSU rDNA sequence data (Fig. 1) suitably placed the present taxon in *Agaricus* sect. *Rubricosi* R.L. Zhao with strong ML and BYPP support value (100% MLBS, 1.00 PP, Fig. 1).

Among morphologically related taxa, *Agaricus variabilicolor* R.L. Zhao differs by having a light brown cap that is not darker at the disc of the pileus, a floccose stipe surface, and much smaller basidiospores (4.1–5.9 × 2.4–3.9 μm). *Agaricus kunmingensis* R.L. Zhao has a pileus the surface of which discolors reddish brown on bruising, and much smaller sized basidiospores (4.1–5.3 × 2.7–3.3 μm). The new species, *Agaricus tumingensis*, exhibits a positive KOH reaction, resulting in a slight reddish-brown discoloration on the pileus and stipe surfaces. Additionally, Schäffer’s reaction is negative on the pileus. The basidiomata of *A*. *tumingensis* consistently develop a reddish-brown upon touching, cutting, and exposure to KOH, distinguishing it from *A*. *variabilicolor* and *A*. dolichopus, where discoloration is weak or indistinct. In contrast, *A*. *kunmingensis* shows a pronounced reddish-brown discoloration in all parts^12^ (Fig. 4; Table 3).

**Table 3:** Morphological comparison of *Agaricus kunmingensis*, *A*. *tumlingensis*, *A*. *dolichopus*, and *A*. *variabilicolor*.

Characters	*A. kunmingensis* (ZRL2012007)	*A. tumlingensis* (CUH AM752)	*A. dolichopus* (ZRL2014120)	*A. variabilicolor* (ZRL4007)
Pileus	20–35 mm diam., campanulate, convex to subumbonate.	33–86 mm diam., convex to broadly convex.	40–45 mm diam., plane, surface dry, covered by fibrillose.	35–80 mm diam., hemispherical, expanding convex.
Stipe surface	Smooth or slightly fibrillose, white.	Covered by dense squamules, gradually decreasing towards the apex.	Smooth or slightly fibrillose, white.	Smooth above the annulus, and scabby or scurfy below the annulus.
Discoloration	Discolouring reddish brown on touching	Turning pastel red to greyish red on bruising	Not discolouring or slightly red on touching.	Not discolouring on touching.
Basidiospores	4.1–5.3 × 2.7–3.3 μm.	5.5–7.5 × 3.5–4.0 μm.	4.5–5.4 × 2.9–3.7 μm.	4.1–5.9 × 2.4–3.9 μm.
Cheilocystidia	14–25 × 8–16 μm.	15–34 × 9–17 μm.	10–21 × 10–14 μm.	9–24 × 7–14 μm.
KOH reaction	Not distinct or slightly reddish brown.	The color turns slightly reddish-brown on the pileus and stipe surfaces.	Not distinct or yellow.	Reddish-brown or not on the surface of the pileus, with no colour change on the stipe.
Schäffer’s reaction	Negative.	Negative.	Negative.	Negative.

**Fig. 5:**
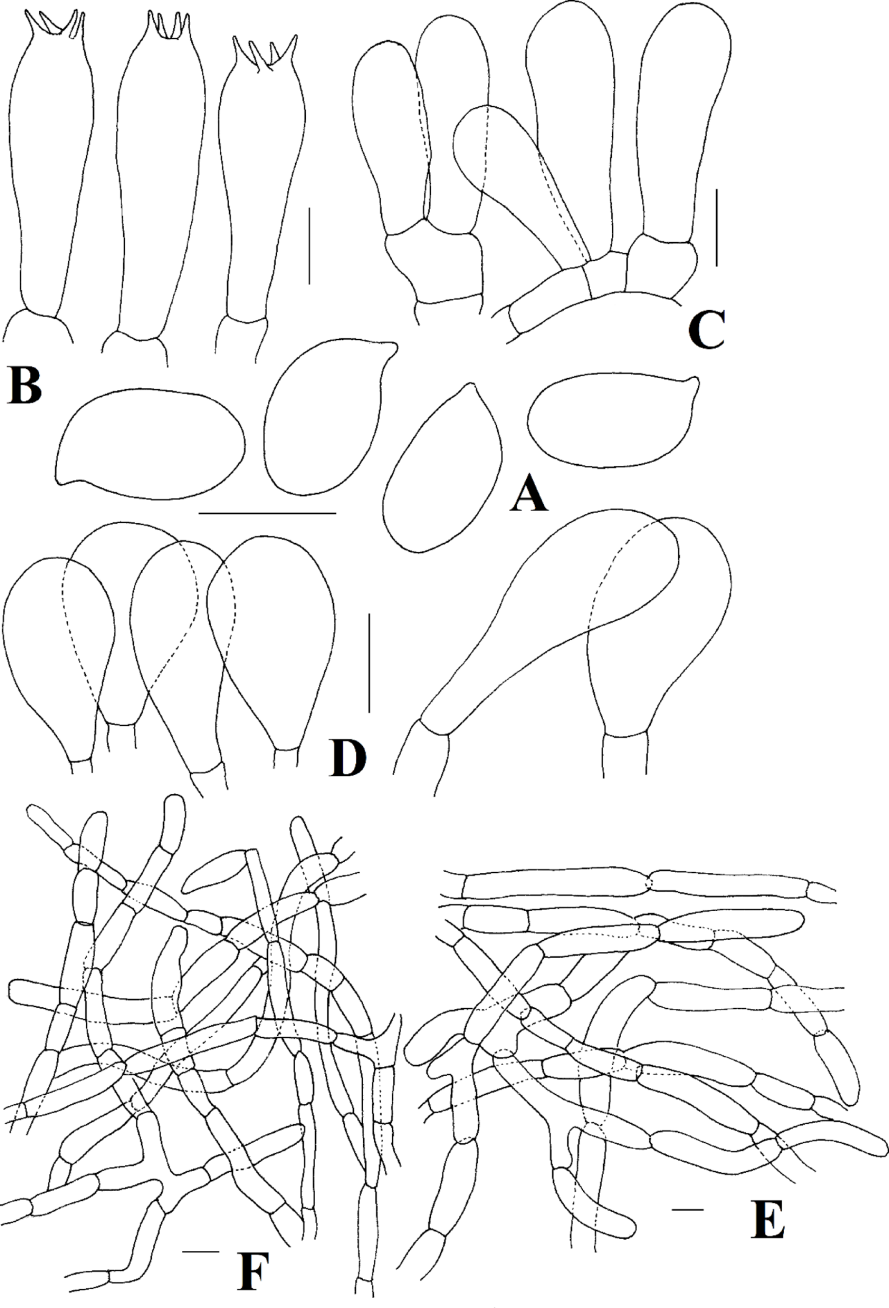
*Agaricus philippei*. (**A**) Basidiospores (**B**) basidia (**C**) basidioles (**D**) cheilocystidia (**E**) pileipellis hyphae (**F**) annulus hyphae. Scale bars: (**A**–**D**) = 5 μm, (**E**–**F**) = 10 μm. Drawings by: E. Tarafder.

*Agaricus philippei* E. Tarafder & K. Acharya, *sp. nov*. Figures 2E–F and 5.

Index Fungorum IF902822.

*Typification*: INDIA: West Bengal, Kalimpong District, Lingsey Village, 27°9′46.79′′ N, 88°40′21.96′′ E, alt. 1119.0 m asl., 18 May 2018, Entaj-53, E. Tarafder & J. Tamang, (holotype CUH AM754). GenBank: ITS = OM654926; LSU = PV436875.

*Diagnosis* Differs from *Agaricus dolichopus* by the presence of dark brown appressed squamules on the pileus surface, ellipsoid to elongate smaller basidiospores 4.5–5.5 × 2.5–3.0 μm and Q = 1.8 on average and cheilocystidia measuring (11–21 × 7–14 μm) and distinctive nrITS and LSU rDNA sequences and position in the phylogram.

*Etymology* The specific epithet “*philippei* (Lat.)” is in honor of the late Dr. Philippe Callac, a renowned expert on *Agaricus* who worked at the French National Institute for Agriculture, Food, and Environment (INRAE), France.

*Morphological characteristics* Basidiomata medium sized. Pileus 55–83 mm diam., broadly convex to plano-convex on maturity, entirely covered with appressed squamules, denser at the centre, dark brown to blackish (6F6) at the umbo, elsewhere dark brown (6D7) on a white to cream background, turning reddish brown on bruising, margin often rimose at maturity, often with traces of partial veilar remnants. Context 2–3 mm thick at the centre, gradually thinner towards margin, white. Lamellae free, 4–6 mm broad, close, with 2–3 series of lamellulae, pinkish (11A2) when young, brown to dark brown at maturity, edge concolorous, regular. Stipe 35–40 × 5–9 mm, central, cylindrical, gradually broader towards the base (12 mm), white to cream or light brown, turning reddish brown (9E5, 9D5) on bruising, smooth, context hollow, white to cream. Annulus superous, upper layer radiating from the stipe to pileus margin like a cobweb pattern, lower layer fibrillose, white. Odour indistinct.

*Macrochemical reactions* KOH reaction positive, color turns yellowish on pileus surface. Schäffer’s reaction was negative on the pileus surface.

*Microscopical characteristics* Basidiospores [60/2/1] (3.5–)4.5–5.5(–6.1) × (2.2–)2.5–3.0(–3.5) µm, [Xm = 5.1 ± 0.7 × 2.7 ± 0.2 μm, Q = 1.5–2.0, Qm = 1.8 ± 0.2], ellipsoid to elongate, light brown to brown, smooth, thick-walled, upto 0.5 μm thick. Basidia 15–25 × 5–8 μm, clavate to cylindrical, hyaline, smooth, tetrasterigmate, sterigmata 1.5–2.2 μm long. Basidioles 12–15 × 5–6 μm, clavate to cylindrical, hyaline, smooth. Cheilocystidia 13–32 × 7–14 μm, abundant, clavate, pyriform, smooth, hyaline. Pleurocystidia absent. Pileipellis a cutis composed of hyphae 5–15 μm broad, slightly constricted at the septa, contain brown pigments. Stipitipellis hyphae 6–9 μm broad, hyaline, septate, thin-walled. Annulus composed of hyphae 5–12 μm broad, hyaline, branched, cylindrical, with rounded apex.

*Habit*, *habitat*, *and distribution*: In a group, on humus mixed soil among the grassy fields.

*Additional specimens examined*: INDIA: West Bengal, Kalimpong District, Lingsey Village, 27°9’46.79"N, 88°40’21.96"E, alt. 1119.0 m asl., 28 May 2018, E. Tarafder, CUH AM541. GenBank: CUH AM541 - ITS: PQ396192.

*Notes* The morphological features of the present taxon include a pileus covered by brown to dark brown colored appressed squamules with a pileus margin associated with traces of partial veilar remnants; cylindrical stipe with broader base turning reddish brown on bruising; complex annulus with lower layer fibrillose and upper layer radiating from stipe to pileus margin like a cobweb pattern; ellipsoid to elongate basidiospores (5.1 × 2.6 μm, Figs. 2E-F); and presence of abundant clavate to pyriform cheilocystidia (14–32 × 7–14 μm); and nrITS, LSU rDNA sequence analyses, the present taxon cluster with members of the sect. *Rubricosi* (Fig. 1) signifies its position within that section.

Regarding overall morphology, the present specimen is somewhat similar to taxa such as *Agaricus dolichopus* R.L. Zhao and *A*. *kunmingensis* R.L. Zhao. However, *A*. *dolichopus* R.L. Zhao has a much smaller pileus (40–45 mm) covered with fibrillose squamules shaped triangular with a slightly upturned tip, mostly unchanging stipe surface, and an annulus, the upper layer of which is never arranged in a cobweb pattern^12^ (Table 4). *Agaricus kunmingensis* has a much smaller pileus (20–35 mm) with its surface covered by light brown squamules, ellipsoid basidiospores (Q mean 1.5), and indistinct to slightly red coloration with KOH^12^ (Fig. 5).

**Table 4:** Morphological comparison of *Agaricus kunmingensis*, *A*. *philippei*, *A*. *dolichopus*, and *A*. *variabilicolor*.

Characters	*A. kunmingensis* (ZRL2012007)	*A. philippei* (CUH AM754)	*A. dolichopus* (ZRL2014120)	*A. variabilicolor* (ZRL4007)
Pileus	20–35 mm diam., campanulate, convex to subumbonate.	55–83 mm diam., convex to broadly convex.	40–45 mm diam., plane, surface dry, covered by fibrillose.	35–80 mm diam., hemispherical, expanding convex.
Stipe surface	Smooth or slightly fibrillose, white.	Smooth, white to cream.	Smooth or slightly fibrillose, white.	Smooth above the annulus, and scabby or scurfy below the annulus.
Discoloration	Discolouring reddish brown on touching.	Turning reddish brown on bruising.	Not discolouring or slightly red on touching.	Not discolouring on touching.
Basidiospores	4.1–5.3 × 2.7–3.3 μm.	4.5–5.1 × 2.5–3.0 μm.	4.5–5.4 × 2.9–3.7 μm.	4.1–5.9 × 2.4–3.9 μm.
Cheilocystidia	14–25 × 8–16 μm.	13–32 × 7–14 μm.	10–21 × 10–14 μm.	9–24 × 7–14 μm.
KOH reaction	Not distinct or slightly reddish brown.	Color turns yellowish on the pileus surface.	Not distinct or yellow.	Reddish-brown or not on the surface of the pileus, with no colour change on the stipe.
Schäffer’s reaction	Negative.	Negative.	Negative.	Negative.

## Discussion

The present study identified *Agaricus kunmingensis*, the first record of the species from India, outside of its type locality China^12^. Morphologically, members of the sect. *Rubricosi* possess an agaricoid basidiomata with a surface of pileus and stipe discoloring reddish brown on bruising, negative Schäffer’s reaction and KOH reaction indistinct or slightly reddish brown, odor pleasant or phenol-like, annulus double, superous, membranous, and often floccose at the lower side and pyriform cheilocystidia^12^.

In both macro-morphological characteristics and nrITS, LSU rDNA sequence analyses, the Indian collections *A. tumlingensis* and *A. philippei* cluster with members of the sect. *Rubricosi* (Fig. 1) signifies its position within that section. Detailed morphological features, along with phylogenetic studies, support the recognition of *A. tumlingensis* and *A. philippei* as new species. Besides, the clustering of *A. tumlingensis* and *A. philippei* together with the members of the sect. *Rubricosi* (Fig. 1) confirms its systematic position within *A*. subg. *Pseudochitonia* with 98% ML and 1.00 BYPP bootstrap support value (Fig. 1).

Phylogenetically, *Agaricus tumlingensis* is closely related to *A*. *variabilicolor*, *A*. *dolichopus*, and *A*. *kunmingensis* with strong support (100% ML and 1.00 BYPP bootstrap support value; Fig. 1). Despite this close relationship, *A*. *dolichopus* differs from *A*. *tumlingensis* by its small-sized basidiomata and basidiospores^12^. *Agaricus variabilicolor* has a light brown cap that lacks a distinctly darker disc and a stipe with a floccose surface. *Agaricus kunmingensis* exhibits a distinct reddish-brown discoloration in all parts upon bruising, and has much smaller basidiospores^12^. Similarly, *Agaricus philippei* is phylogenetically related to *A*. *variabilicolor*, *A*. *dolichopus*, and *A*. *kunmingensis* with 98% ML and 1.00 BYPP bootstrap support value (Fig. 1). However, *A. variabilicolor* can be distinguished from *A*. *philippei* by its light brown pileus, which is not darker at the disc of the pileus and has a floccose stipe surface, smaller basidiospores, and cheilocystidia^12^. *Agaricus dolichopus* differs from *A*. *philippei* by its small-sized basidiomata, smaller basidiospores, and pyriform to broad clavate cheilocystidia^12^. Additionally, *Agaricus kunmingensis* has a distinct reddish-brown discoloration in all parts on bruising, and much smaller sized basidiospores^12^. Moreover, the phylogenetic analysis of the nrITS and LSU rDNA sequences confirmed that *A*. *tumlingensis* and *A*. *philippei* are clearly distinguishable from other previously described and sequenced *Agaricus* species of the sect. *Rubricosi* forms a close sister clade to sections *Nigrobrunnescentes*, *Bohusia*, and *Sanguinolenti*, and all are reddish discoloring species. Notably, all species within the sect. *Rubricosi* are exclusively native to the tropical and subtropical ecosystems^12^.

The discovery of *Agaricus tumlingensis*, *Agaricus philippei*, and the first record of *Agaricus kunmingensis* in India highlight the fungal biodiversity of the region.

## Data Availability

Sequence data generated for the present study have been deposited in GenBank with the accession numbers can be found below: https://www.ncbi.nlm.nih.gov/genbank, CUH AM751 (ITS: OM279500; LSU: PV341363); CUH AM752 (ITS: OM654924; LSU: PV341365); CUH AM753 (ITS: OM654925; LSU: PV341366); CUH AM754 (ITS: OM654926 LSU: PV436875); CUH AM541 (ITS: PQ396192). All of the data that support the findings of this study are available in the main text.
